# HACS1 signaling adaptor protein recognizes a motif in the paired immunoglobulin receptor B cytoplasmic domain

**DOI:** 10.1038/s42003-020-01397-z

**Published:** 2020-11-13

**Authors:** Jamie J. Kwan, Sladjana Slavkovic, Michael Piazza, Dingyan Wang, Thorsten Dieckmann, Philip E. Johnson, Xiao-Yan Wen, Logan W. Donaldson

**Affiliations:** 1 Department of Biology, York University, 4700 Keele Street, Toronto, M3J 1P3 ON Canada; 2 Department of Chemistry, York University, 4700 Keele Street, Toronto, M3J 1P3 ON Canada; 3 Department of Chemistry, University of Waterloo, 200 University Avenue, Waterloo, N2L 3G1 ON Canada; 4 Keenan Research Centre for Biomedical Science, St. Michael’s Hospital, 30 Bond Street, Toronto, M5B 1W8 ON Canada

**Keywords:** Phosphoproteins, Solution-state NMR

## Abstract

Hematopoietic adaptor containing SH3 and SAM domains-1 (HACS1) is a signaling protein with two juxtaposed protein–protein interaction domains and an intrinsically unstructured region that spans half the sequence. Here, we describe the interaction between the HACS1 SH3 domain and a sequence near the third immunoreceptor tyrosine-based inhibition motif (ITIM3) of the paired immunoglobulin receptor B (PIRB). From surface plasmon resonance binding assays using a mouse and human PIRB ITIM3 phosphopeptides as ligands, the HACS1 SH3 domain and SHP2 N-terminal SH2 domain demonstrated comparable affinities in the micromolar range. Since the PIRB ITIM3 sequence represents an atypical ligand for an SH3 domain, we determined the NMR structure of the HACS1 SH3 domain and performed a chemical shift mapping study. This study showed that the binding site on the HACS1 SH3 domain for PIRB shares many of the same amino acids found in a canonical binding cleft normally associated with polyproline ligands. Molecular modeling suggests that the respective binding sites in PIRB ITIM3 for the HACS1 SH3 domain and the SHP2 SH2 domain are too close to permit simultaneous binding. As a result, the HACS1-PIRB partnership has the potential to amalgamate signaling pathways that influence both immune and neuronal cell fate.

## Abstract (web summary)

Kwan et al. show the interaction between the HACS1 SH3 domain and a sequence near the third immunoreceptor tyrosine-based inhibition motif of the Paired immunoglobulin receptor B (PIRB). This study suggests that the HACS1-PIRB partnership has the potential to unite signaling pathways that regulate both immune and neuronal cell fate.

## Introduction

HACS1 (SLy2, SAMSN1, NASH1, SASH2) is a signaling adaptor protein that was first associated with leukemia. Since that discovery, HACS1 has been recognized further in blood vessel development^1^ and the progression of cancers with aggressive phenotypes^2,3^. By virtue of its location on chromosome 21, there is interest in exploring the role of HACS1 in Down’s syndrome^4,5^ and late-onset Alzheimer’s disease^5,6^. Sequence analysis of human HACS1 and its homolog, HACS2/SLy1^7–11^, reveal juxtaposed SAM^12^ and SH3^13^ domains that are well-known mediators of protein–protein interactions. Since over half of HACS1 is predicted to be intrinsically disordered, this adaptor protein has the potential to serve as a platform for many yet to be discovered protein partners and post-translational modifications. This report presents a biochemical and structural study of an interaction between the HACS1 SH3 domain and the cytosolic region of the Paired immunoglobulin receptor B (PIRB) receptor that has not been explored since its original identification by yeast two-hybrid methods.

Positive signals arising from antigen receptors are antagonized by HACS1 and PIRB^14^. Thus, if either HACS1 or PIRB is inactivated, the immune system becomes hypersensitive. In HACS^15^ and PIRB knockout mice^16^, hypersensitivity is manifested by enhanced T-helper cell response and increased cytokine signaling. Since HACS1 and PIRB mutants have similar phenotypes, it supports the idea that their interaction may be required in certain immune signaling pathways.

The murine PIRB receptor and its five human leukocyte immunoglobulin-like receptor (LIRLB) orthologs have received considerable research interest not only to due their participation in immune signaling, but also in infection^17^, platelet activation^18^, brain repair^19–24^, and Alzheimer’s disease^25^. As a result of this interest, HACS1 represents one relatively under-investigated member of a large repertoire of PIRB/LILRB protein partners that include major histocompatibility complex related proteins^26^, and oligomeric ß-amyloid^25^.

The PIRB cytoplasmic domain contains four ITIMs (intracellular tyrosine-based inhibitory motifs) which, upon phosphorylation, are able to recruit SHP family phosphatases^27^. Normally, SHP phosphatases are autoinhibited until they are phosphorylated themselves or bind a partner like PIRB. Once activated, SHP phosphatases inactivate downstream kinases including Syk, Btk, and PLC-γ that act as sentinels for pathways involved in proliferation, survival, and the production of cytokines in the immune response. The discovery of HACS1 as a protein partner binding to PIRB therefore creates a new mode of regulation at or near ITIMs that may either complement or compete with SHP family proteins.

In this present investigation, we have used a combination of nuclear magnetic resonance (NMR) spectroscopy, isothermal titration calorimetry, differential scanning calorimetry, circular dichroism spectroscopy, and surface plasmon resonance methods to identify new structural and biochemical features of the HACS1-PIRB interaction. An amalgamation of these experimental observations with molecular modeling suggests that the PIRB-HACS1 relationship represents an example of an atypical ligand binding an SH3 domain with a correspondingly atypical binding cleft. Molecular modeling is also used to explore the potential for cooperation or competition with SHP family phosphatases.

## Results

### Structure of the HACS1 SH3 domain

Human and mouse HACS1 are 86% identical over 372 aa. When the SH3 domain is considered separately, the human and mouse sequences are essentially identical, differing only by one conservative substitution of arginine for lysine (Figure S1). Throughout this study, a number of human HACS1 SH3 domain protein fragments were expressed with a 6xHis-tag or as a 6xHis-MBP fusion protein. The boundaries chosen were consistent with sequence comparisons with other SH3 domains. A 0.2 mM sample of reduced, 6xHis-tagged SH3 domain assayed by multi-angle laser light scattering revealed that protein was monomeric under the pH and salt conditions used throughout this study (Figure S2).

**Fig. 1:**
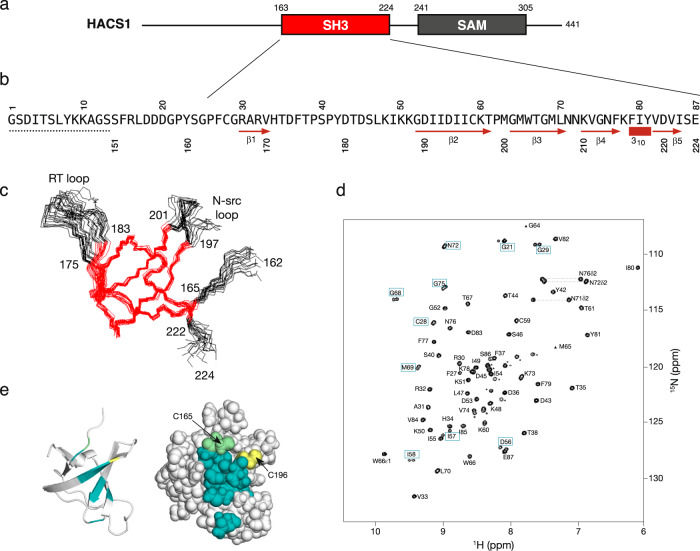
Domain organization and structure of the HACS1 SH3 domain. (**a**) The SH3 and SAM domains are separated by a linker region of seventeen residues. (**b**) The protein fragment used for structural studies includes thirteen non-native residues (dotted line). Secondary structures deduced from the NMR solution structure are indicated below the sequence. (**c**) Superposition of twenty lowest energy structures after refinement. The highest precision regions are colored red. The numbering follows the human HACS1 sequence. (**d**) ^1^H-^15^N HSQC spectrum of the HACS1 SH3 domain. Unassigned resonances are indicated with an asterisk. Amide resonance pairs are indicated by rectangles. (**e**) Amide resonance pair map to one surface patch on the SH3 domain. Two cysteines, one observed as a resonance pair (green) and one nearby (yellow) do not form intramolecular disulfide bonds despite their proximity.

The NMR structure of the human HACS1 SH3 domain was determined using a combination of NOE distance restraints, torsion angle restraints derived from backbone chemical shifts and amide residual dipolar couplings (Table 1) and was deposited in the Protein Data Bank with the accession number 6UZJ. Backbone and side-chain proton assignments were 96.1% and 90.1% complete, respectively (Table S1 & Figure S3). Inclusion of amide dipolar coupling data improved the quality factor (Q) from 41.0% to 10.8% (Figure S4). Omitting the non-native sequence that remained after proteolytic removal of the MBP carrier protein, the first eighteen amino acids of the protein fragment were unstructured; thus, the minimal folded SH3 domain spans residues 168–222 in HACS1 (Fig. 1a and b). The RT-loop between β1 and β2 is less precisely defined as shown by the ensemble of the twenty lowest energy structures in Fig. 1c. While the RT-loop was fully assigned, few medium- and long-range NOEs were observed that would lead to a unique structure solution for this region. From a relaxation study at 700 MHz (Figure S5), ^15^N-T2 relaxation times in portions of the RT-loop were larger than the average of 130 ms and had larger associated errors associated with fits to a monoexponential decay model. Heteronuclear ^15^N{^1^H} NOEs for Y179 and T181 were lower than the average value of 0.81. The ^15^N-T_1_ relaxation times for the RT-loop, in contrast, were consistent with the average value of 620 ms. Taken together, the ^15^N relaxation data suggests that the RT-loop is dynamic relative to the rest of the SH3 domain. A rotational correlation time of 5.2 ns was determined from a global fit of the relaxation data and corroborates the conclusions of the multi-angle laser light scattering study that the HACS1 SH3 is monomeric.

**Table 1:** NMR and refinement statistics.

	HACS1 SH3 domain
**NMR distance and dihedral constraints**	
Distance constraints	
Total NOE	1148
Intra-residue	495
Inter-residue	
Sequential (|*i* – *j* | = 1)	245
Medium-range (|*i* – *j* | < 4)	75
Long-range (|*i* – *j* | > 5)	333
Hydrogen bond restraints a	9
Hydrogen bonds inferred b	25
Total dihedral angle restraints	
ϕ	61
ψ	61
Total RDCs	49
Q before refinement with RDCs (%)	41
Q after refinement with RDCs (%)	11
**Structure statistics**	
Violations (mean and s.d.)	
Distance constraints (Å)	0.0349 ± 0.0023
Dihedral angle constraints (°)	0.4255 ± 0.0153
Deviations from idealized geometry	
Bond lengths (Å)	0.0025 ± 0.0005
Bond angles (°)	0.4255 ± 0.0153
Impropers (°)	0.3185 ± 0.0134
Average pairwise r.m.s. deviation among 20 structures (Å)	
Heavy	0.32
Backbone	1.16

a hydrogen bond restraints submitted to the HBDA module of XPLOR 2.52.

b hydrogen bonds inferred by the HBDB module of XPLOR 2.52.

The structure of murine SAMSN1/HACS1 has been solved as part of an NMR-focused structural proteomics program (PDB: 2EBP) but has not been described in the literature. The two structures superimpose with a 1.7 Å RMSD across all amino acids. When the RT and N-Src loops are removed from the comparison, the backbone RMSD is 1.0 Å.

A number of resonance pairs were observed in the ^1^H-^15^N HSQC spectrum of the HACS1 SH3 domain (Fig. 1d). Originally, we suspected that two closely positioned cysteines were promoting intermolecular dimer formation. However, these resonance pairs were still observed in reduced samples. A temperature series of ^1^H-^15^N HSQC spectra from 5 to 40 °C was acquired and, in each case, resonance pairs were still observed. As shown in Fig. 1e, the resonance pairs map to a solvent-exposed face of the central ß-sheet that is connected by an extensive hydrogen-bonding network. Although we cannot provide an explanation for the conformational change contributing to these resonance pairs, we speculate that it is concerted in some way. By differential scanning calorimetry, the HACS1 SH3 domain thermally denatured in an irreversible manner at 47.5 °C with an enthalpy of 515 ± 7 kJ/mol and maximal unfolding free energy of 24 kJ/mol (Figure S6). SH3 domains demonstrate a wide range of thermostabilities^28^ in the special case of Drk SH3, an equilibrium of folded and unfolded states are observed at room temperature^29^.

### Mapping the HACS1-PIRB interaction

**Fig. 2:**
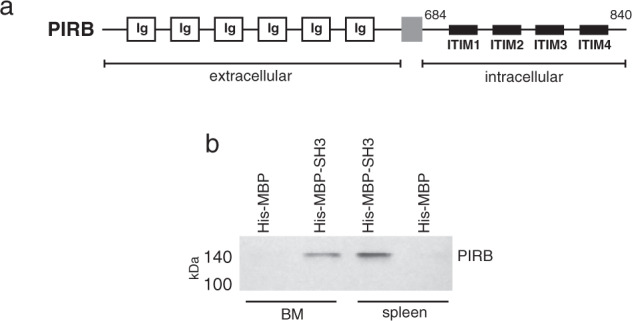
The HACS1–PIRB interaction. (**a**) Schematic representation of the PIRB receptor. The cytoplasmic region with four ITIM repeats spans approximately 190 residues. (**b**) Western detection of the HACS1-PIRB interaction. A pulldown was performed with immobilized HACS1 SH3 domain fragments fused to maltose-binding protein (MBP) and extracts of mouse bone marrow (BM) or spleen. Chemiluminescent detection of PIRB was achieved with a mouse anti-PIRB antibody.

PIRB is a receptor consisting of six immunoglobulins (Ig) repeats in its extracellular segment, a thirty amino acid hydrophobic transmembrane segment, and a 190 aa. cytosolic domain with four immunoreceptor tyrosine-based inhibition motifs (ITIMs). Each ITIM is consistent with the consensus sequence [SIVL]-Y-xx-[IVL] (Fig. 2a). When PIRB is activated through extracellular ligand binding, the ITIMs are phosphorylated and recognized by the tandem SH2 domains of the SHP-1 and SHP-2 protein tyrosine phosphatases^30,31^. ITIMs are balanced by a reciprocal set of immunoreceptor tyrosine-based activation motifs (ITAMs) that in B-cell receptors, for example, are phosphorylated by tyrosine kinases such as Syk that promote cell maturation^32^. The interaction between HACS1 and PIRB cytosolic region may bypass SHP phosphates and introduce a new junction in signaling pathways governing immune- and neuronal cell fate. Building upon a yeast two-hybrid study that first identified a possible HACS1-PIRB partnership, we extended this observation by demonstrating that the HACS1 SH3 domain was sufficient for direct interaction with PIRB. In Fig. 2b, purified 6xHis-MBP tagged HACS1-SH3 domain pulled down PIRB from immune cell lysates derived from mouse bone marrow and spleen while a purified 6xHis-MBP could not. A full blot is provided in Figure S7.

**Fig. 3:**
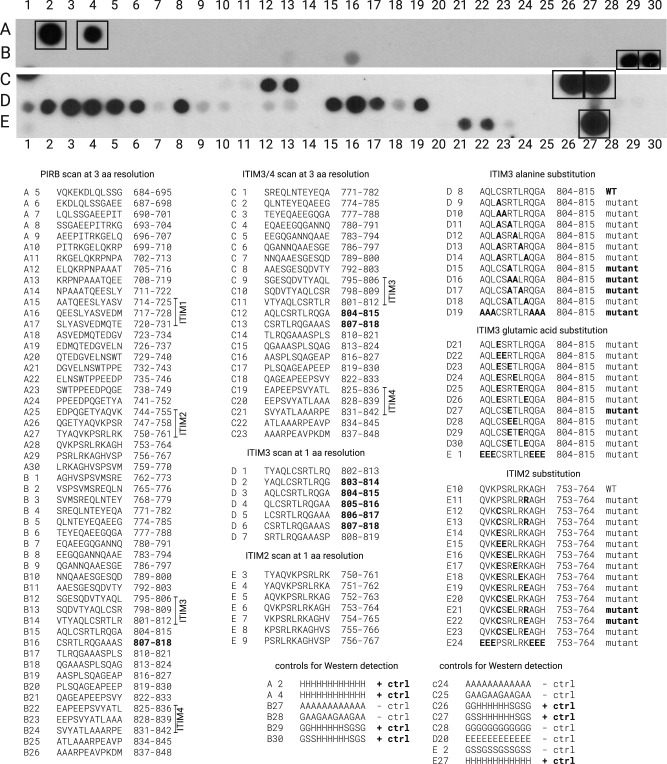
Peptide array analysis. A series of 12-mers spanning the entire region of the intracellular region of PIRB were used to identify the binding site for the HACS1 SH3 domain. Detection was performed by chemiluminescent antibody-based detection of a 6xHis tag on a purified SH3 domain probe. Strong signals are denoted in the legend as boldface. A series of alanine and glutamic acid substitutions were also performed (rows D and E). Positive controls are indicated by squares on the blot.

An array of 12-mer peptides spanning the entire PIRB cytosolic domain at a three amino acid resolution was synthesized on a cellulose membrane and challenged with a 6xHis-tagged HACS SH3 domain. As many SH3 domains participate in low-affinity interactions with their ligands, the blot was probed with protein at a concentration of 5 µM. After stringent washing and chemiluminescent detection with an antibody against the 6xHis tag on the protein, a series of positive binding signals were observed overlapping ITIM3, but not the other ITIMs (Fig. 3). The experiment was then repeated with one amino acid resolution across the region around ITIM3. From these binding experiments, a peptide corresponding to the murine sequence CSRTLRQGAAAAS consistently produced a strong signal. It is worth noting that we observed another signal in the vicinity of ITIM3 (DVTYAQLCSRTL). However, this signal was weaker and therefore, less confidence was ascribed to it. Overall, the PIRB motif appears to represent a new SH3 binding motif. A comprehensive binding landscape was recently described for over one hundred SH3 domains and among the proteins in the set, many non-canonical ligands were discovered^33^. The HACS1 SH3 domain; however, was not among the proteins that was surveyed.

A second peptide blot was synthesized and probed to refine the HACS1–PIRB interaction further. From a peptide walk of the ITIM3 region at a one amino acid resolution, the strongest signals all contained the six amino acid sequence, CSRTLR. Substitution at three amino acids flanking each side of this core sequence with alanine or glutamic acid diminished binding suggesting that the flanking sequences support binding by making auxiliary contacts or supporting the formation of a specific structure. Further substitution of the central amino acids (CSRTLR) to alanine was tolerated. In contrast, the substitution of cysteine at either to alanine (spot D9 in Fig. 3) or glutamic acid (spot D21 in Fig. 3) eliminated binding altogether.

Next, we examined the importance of two charged amino acids in the binding site. In ITIM3, the two amino acids are both arginine (CSRTLR). If both arginines were substituted with either alanine (CSATLA; spot D18 in Fig. 3) or glutamic acid (CSETLE; spot D30 in Fig. 3), binding was abolished. In contrast, if the first arginine alone was substituted with either alanine (CSATLR; spot D15 in Fig. 3) or glutamic acid (CSETLR; spot D27 in Fig. 3), binding was retained. While these data initially suggested that the second arginine contributes important contacts to HACS1, a follow-up substitution analysis of ITIM2 revealed that the contribution of two charged sites with HACS1 is not as straightforward as we originally believed.

Unlike the CSRTLR sequence in ITIM3, the six central amino acids of ITIM2 are PSRLRK. In particular, the first position (PSRLRK) deviates from cysteine that was shown to be an important determinant of binding in ITIM3. An ITIM2 sequence substituted with cysteine at the first position (CSRLRK, spot E12 in Fig. 3) did not bind HACS1 despite this sequence retaining the two basic amino acids that appeared to be important for binding. One additional change was made to this sequence, replacing the lysine with arginine to be consistent with ITIM3 (CSRLRR; spot E13 in Fig. 3). However, this sequence did not bind ITIM3, either. In contrast, the ITIM3 variants CSETLR (spot D27 in Fig. 3) and CSELRR (spot E21 in Fig. 3) bound HACS1 in addition to an ITIM2 variant CSRLRE (spot E22 in Fig. 3) where the two charged amino acids were switched.

Amalgamating the peptide array data, the HACS1 SH3 domain appears to prefer binding peptides with the consensus sequence C-x-[R/E]-Φ-Φ-[E/R], where Φ is any aliphatic amino acid. We attempted to improve this consensus by comparing the murine PIRB/LILRB3 sequence with the orthologous rat and human sequences. While the rat ITIM3 sequence is identical to the mouse, the human ITIM3 sequence is HSLTLR. Thus, the human sequence modifies the HACS1 consensus binding site to [C/H]-S-[Φ/R/E]-Φ-Φ-[E/R].

### Chemical shift surface mapping the PIRB ligand on the HACS1 SH3 domain

**Fig. 4:**
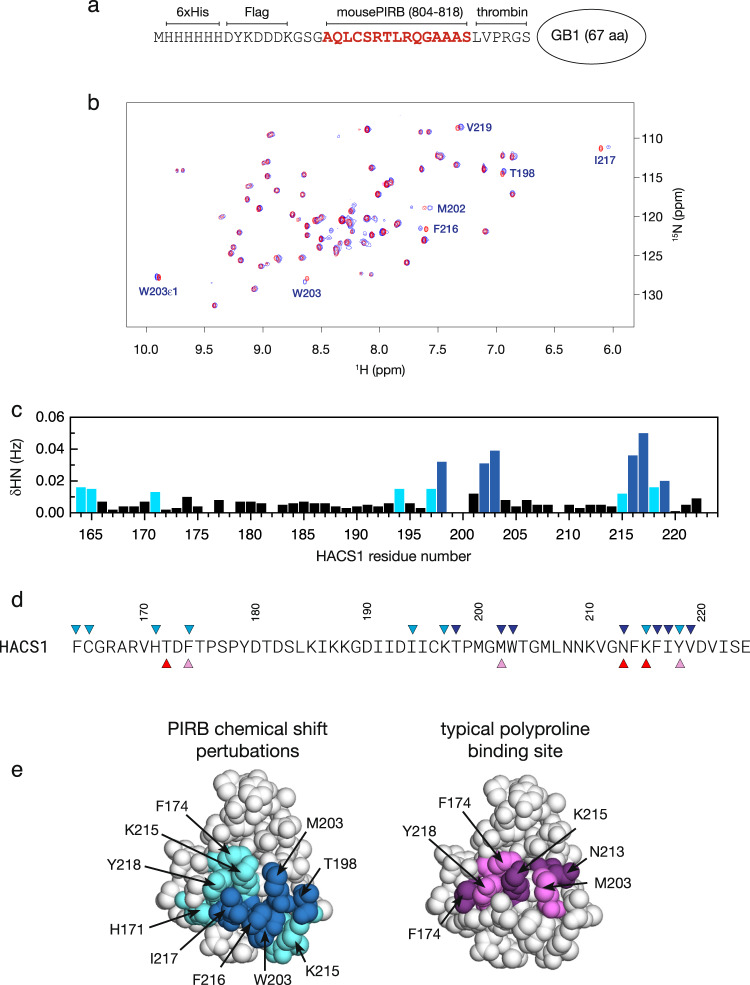
Mapping the PIRB binding site on the HACS1 SH3 domain. (**a**) A titration was performed with a soluble fusion protein consisting of 6xHis affinity tag, Flag epitope tag, and murine PIRB ITIM3 sequence fused amino-terminally to the Protein G B1 domain (GB1) within an intervening thrombin cleavage site. (**b**) Superimposed ^1^H-^15^N HSQC spectra of ^15^N-labeled HACS1 SH3 domain (red) and the HACS1 SH3 domain with a ten-fold excess of a PIRB-GB1 protein (blue). Selected peaks with the largest amide chemical shift perturbations (CSPs) are indicated. (**c**) A plot of weighted ^1^H/^15^N amide CSPs. Light and dark blue shading indicate CSPs that exceed a one and two standard deviation threshold, respectively. (**d**) Triangles above the HACS1 sequence coincide with CSPs of the previous panel. Triangles below sequence indicate residues that comprise a typical peptide binding cleft on an SH3 domain. (**e**) CSPs from the PIRB-GB1 titration were mapped onto the NMR structure of the HACS1 SH3 domain following the color scheme from the previous panels. For comparison, a typical binding cleft is shown with the same color scheme as the previous panel.

To map the binding site of the PIRB ITIM3 sequence on the surface of the HACS1 SH3 domain, we compared the ^1^H,^15^N-HSQC spectra of the HACS1 SH3 domain in the absence and presence of a ten-fold excess of PIRB ITIM3 sequence fused amino-terminally to the 57 aa. Protein G B1 domain (GB1, Fig. 4a). In addition to providing a way to make PIRB ITIM3 ligands recombinantly, the PIRB-GB1 hybrid protein was very soluble at high concentrations (>1 mM) required for NMR-based experiments and calorimetry. Amide chemical shift perturbations (CSPs) in the spectra depicted in Fig. 4b are plotted as a histogram in Fig. 4c and colored according to the number of standard deviations from the average value. These CSPs, in turn, were mapped to the NMR structure of the SH3 domain to identify a potential PIRB ITIM3 binding site. A comparison of the PIRB binding site identified by CSPs and the typical SH3 domain binding cleft (Fig. 4d) reveals a considerable degree of overlap although the PIRB binding site appears to require additional contributions (Fig. 4e). The most striking difference between the PIRB binding site on HACS1 and a typical SH3 binding cleft is at K215. This amino acid is positioned in the middle of the cleft, normally where an aromatic amino acid would be expected. As a result, the charge and steric interference caused by a lysine would likely preclude the binding of any proline-rich ligand. Thus, HACS1 SH3 domain is among eleven structures that have been solved to date with an atypical ligand binding cleft^34^.

### The affinity of PIRB ITIM3 for SH2- and SH3-domain peptides

**Fig. 5:**
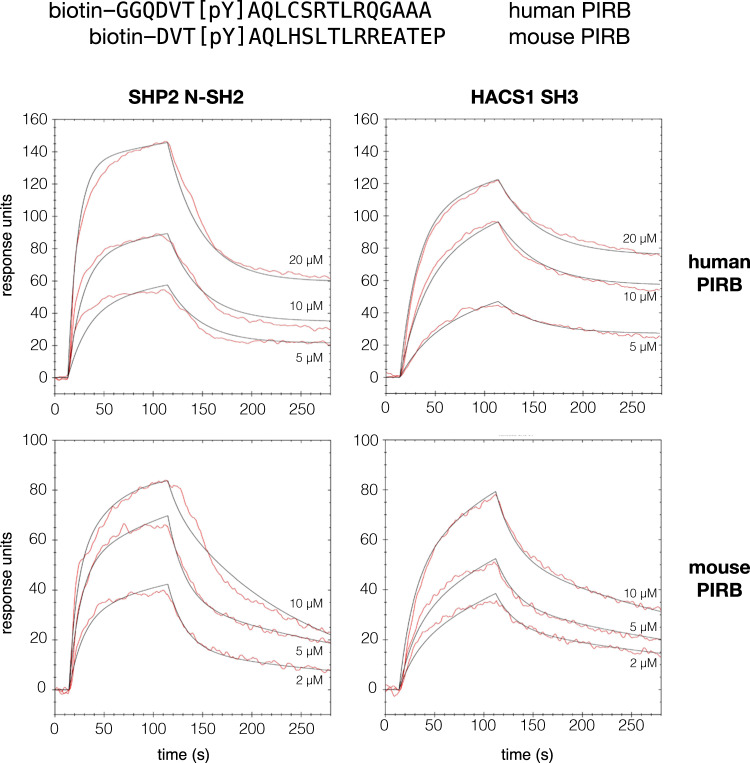
Binding affinities of the HACS1 SH3 domain and the SHP1 SH2 domain for ITIM3 sequences. A biotinylated human or murine derived phosphopeptide was immobilized onto a streptavidin-coated gold sensor chip for a surface plasmon resonance-based assay. On-rates were determined by applying an analyte solution of purified 6xHis tagged HACS1 SH3 domain or SHP1 N-SH2 domain at the indicated concentrations. Off-rates were determined by switching to an application of buffer alone at 120 s. Curve fits were made according to a 1:1 interaction model.

The region of PIRB ITIM3 that interacts with the HACS1 SH3 domain is near a regulatory tyrosine. When this tyrosine is phosphorylated, ITIM3 becomes a ligand for the two SH2 domains of SHP2 phosphatase, differentiated by the names, SH2(N) and SH2(C). The affinities of the HACS1 SH3 domain and SHP2 SH2-N domain for PIRB were determined using surface plasmon resonance-based assay. Biotinylated murine and human phosphopeptides were immobilized on a streptavidin-coated gold sensor and varying concentrations of the protein analyte solutions were applied and then washed from the sensor at a controlled flow rate. On- and off-rates were simultaneously fit to the resulting sensorgrams and a dissociation constant (*K*_*d*_) was determined. As shown in Fig. 5 and summarized in Table 2, the SHP2 SH2(N) domain and HACS SH3 domain had the same affinities of ~7 µM for human PIRB phosopho-ITIM3. The affinity of the HACS SH3 domain for the murine sequence was weaker at 15.9 µM.

**Table 2:** Kinetic data and affinities of the HACS1 SH3 domain and the SHP2 SH2-N domain from SPR measurements against murine and human PIRB phosphopeptides.

protein	ligand	*k*_*on*_ (M^−1^s^-1^)	*k*_*off*_ (s^-1^)	*K*_*d*_ (M)
SHP2 SH2-N	murine PIRB	6.8 × 10^3^	5.0 × 10^−2^	7.4 × 10^−6^
SHP2 SH2-N	human PIRB	3.8 × 10^3^	2.6 × 10^−2^	6.7 × 10^−6^
HACS1 SH3	murine PIRB	2.4 × 10^3^	3.7 × 10^−2^	15.9 × 10^−6^
HACS1 SH3	human PIRB	1.9 × 10^3^	1.7 × 10^−2^	8.7 × 10^−6^

Isothermal titration calorimetry served as a complementary approach to determine the affinity of HACS1 for PIRB. According to this method, an approximately ten-fold excess of murine PIRB-GB1 fusion protein was gradually injected into a solution of 6xHis-tagged HACS1 SH3 domain and the heat released upon binding was measured. After subtracting the heat of dilution, an affinity of 15.9 µM was determined with a 1:1 stoichiometry, consistent with the SPR study using immobilized phosphopeptides (Figure S8). Furthermore, this study demonstrates that phosphorylation does not affect the binding of the HACS1 SH3 domain to PIRB. This result was expected since phosphotyrosine is outside the region identified by the peptide array.

The pY+4 position is an important determinant of affinity for both the SH2-N and SH2-C domains^35^. When an aromatic or bulky aliphatic ligand occupies the pY+4 site, typical affinities observed are in the range of 0.1–1.0 µM. Amino acids that deviate from this preference may contribute up to ten-fold decrease in affinity. With leucine occupying the pY+4 position in the murine and human PIRB ITIM3 (pYAQL), the observed affinity of 7 µM would be consistent with the prediction that ITIM3 would be a low-affinity SHP2 SH2 domain ligand.

### Molecular modeling of the relationship between HACS1 and SHP1/2

**Fig. 6:**
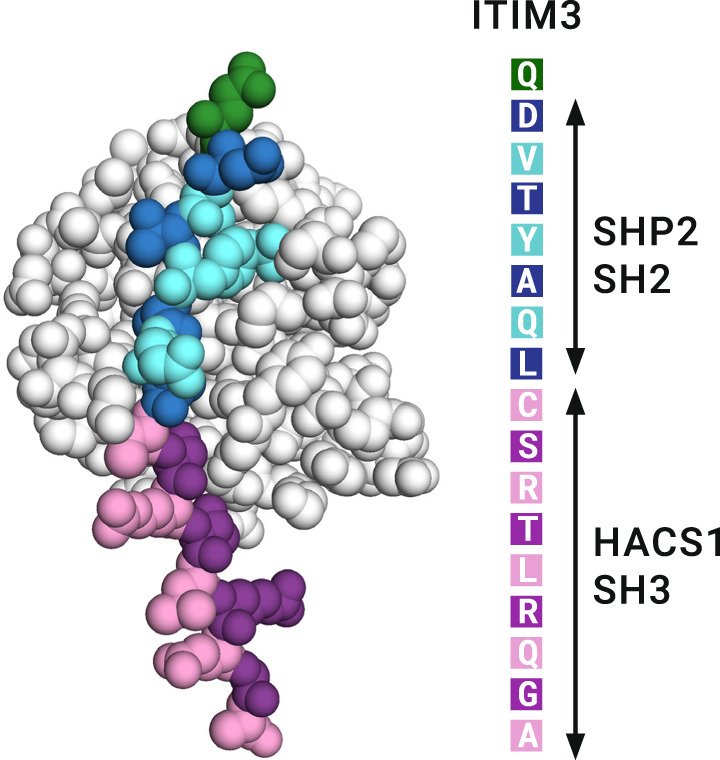
A molecular model of the SHP2 SH2 domain with the ITIM3 region of PIRB. To distinguish the regions of ITIM3 that engage SHP1 and HACS1, the peptide is colored blue and magenta, respectively. One amino acid in the model that is outside the SHP2 and HACS1 recognition region is colored green. The sequence CSR makes contacts with SHP2 SH2 domain in this model and also comprises the central portion of the HACS SH3 domain recognition site.

A molecular model of the SHP2/PIRB interaction was made to investigate the possibility that an SH2 domain and the HACS1 SH3 domain could be bind simultaneously to PIRB ITIM3. As shown in Fig. 6, the SH2 domain binding cleft contacts two additional amino acids beyond the pYAQL motif, and by doing so, would likely be sufficient to prevent the HACS1 SH3 domain from binding. Likewise, the HACS1 SH3 domain with its comparable affinity, may bind near enough to ITIM3 to compete with SHP family phosphatases. We performed a sequence analysis of the PIRB cytoplasmic region using three programs designed to identify intrinsically unstructured regions^36–38^ and three programs designed to identify secondary structured regions^39–41^. The lack of consensus among the secondary structure prediction programs combined with consistently strong predictions by the intrinsically unstructured region prediction programs suggests that the cytoplasmic region including the ITIMs, serve as a large display site^42^ for the transient binding of protein partners (Figure S9). Since we did not determine the structure of the PIRB-HACS1 interaction, the structure the PIRB ITIM3 region remains unknown. Given the low affinity of the interaction with the HACS1 SH3 domain, it may be beneficial in terms of entropy for the PIRB ITIM3 region to be unstructured in both the free and bound states.

## Discussion

This investigation extends a yeast two-hybrid study that identified the SH3 and SAM domain signaling adaptor protein HACS1 (SLy2) as a protein partner of Paired immunoglobulin receptor B (PIRB). Using a combination of pull-down assays, peptide arrays, and binding assays with purified proteins, we have shown that the interaction is direct and mediated by the SH3 domain of HACS1 and a sequence in the PIRB cytosolic domain that overlaps ITIM3, a well-established SH2 domain binding site. Chemical shift mapping identified a surface on the HACS1 SH3 domain that is known for binding both canonical proline-rich ligands and noncanonical ligands such as the RxxK motif of SLP-76^43^. Compared to other typical SH3 domains, the largest amino acid difference in the HACS1 binding surface is K215 where normally a proline would be found. A survey of ITIM3 variants helped define a consensus binding site of [C/H]-S-[Φ/R/E]-Φ-Φ-[E/R] for the HACS1 SH3 domain. This consensus is instructive, but limited by the fact that we did not perform an exhaustive survey with a peptide library. The presence of at least one basic amino acid in the consensus sequence appears to be critical for binding. In the HACS1 SH3 domain, D180 and D182 in the RT-loop are likely candidates to make ionic bonds with basic amino acids in the PIRB ligand due to their proximity to the binding surface.

The NMR and SEC-MALS analyses indicate that the HACS1 SH3 domain is monomeric over a wide range of concentrations. The SAM domain that closely follows the SH3 domain in HACS1 is monomeric, as well (PDB: 1V38). HACS1 has a homolog SLy1/SASH3 that also participates in immune cell developmental programs, but cannot substitute for HACS1 despite having over 40% sequence identity. These functional distinctions appear to extend to the architecture of SLy1 since its SAM domain forms homodimers^44^. It is worth noting that the way in which the SLy1 SAM domain dimerizes is unique among the myriad of homo- and hetero-oligomeric SAM domain structures that have been described.

In lung cancer^45^ and multiple myeloma^2^, HACS1 is underexpressed, while in glioblastoma^3^, it is overexpressed suggesting that the function of HACS1 adaptor protein is complex. As a result, the identification and characterization of protein partners that work with HACS1 represent an important first step towards understanding its precise role in cancer. In this report, we have demonstrated how the SH3 domain serves one such role through its association with PIRB, although it is important to be aware that the remainder of this adaptor protein is also a platform for numerous partners, including 14–3–3 proteins^46^. Genomics studies have implicated HACS1 in Alzheimer’s disease associated pathways, although its role has not been established^6^. In summary, renewed interest in HACS1 and its relationship with PIRB may reveal new ways how extracellular signals affect innate immunity and synaptic signaling.

**Fig. 7:**
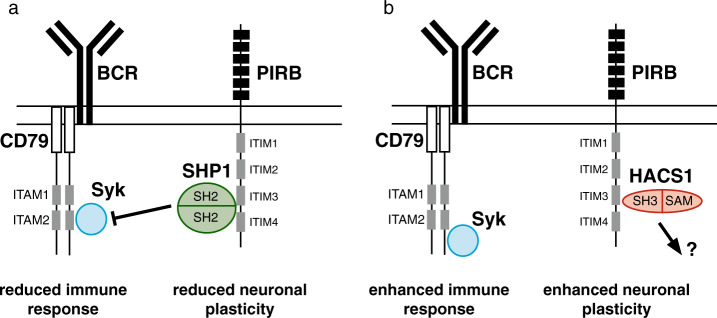
Signaling pathway outcomes involving PIRB and HACS1. (**a**) Experimental evidence to date places PIRB in an antagonistic role towards several kinds of positive receptor signals, including those from B cell antigen receptor (BCR). Antagonism is achieved by the binding of SHP1 phosphatase to phosphorylated ITIMs, that in turn, prevent critical phosphorylations on BCR by kinases including Syk. The SHP1-PIRB relationship also antagonizes pathways associated with neuronal plasticity. (**b**) A similar representation of BCR and PIRB signaling that incorporates the HACS1 relationship presented in this study. If HACS1 can either displace or compete with SHP1 for binding PIRB, Syk may remain activated thereby enabling enhanced BCR signaling and neuronal plasticity. In its adaptor role, HACS1 can bring a different set of regulatory and signaling proteins to PIRB.

Molecular modeling suggests that SHP1 and HACS1 cannot simultaneously bind PIRB. With relatively modest and similar affinities for PIRB, concentration changes of HACS1 and SH2-domain-containing proteins would change the occupancy on PIRB and downstream signaling outcomes. In Fig. 7, we present two scenarios where HACS1 shifts PIRB into a stimulator role from a traditional inhibitory role via SHP1 and SHP2. The outcomes of these two scenarios would be an increase in the sensitivity of the innate immune response and an increase in neuronal plasticity. The specific features of each of these fundamental cellular roles will require a combination of targeted investigations where biochemistry is foremost and global investigations using proteomics methods that can identify the full complement of partners for HACS1 and the PIRB cytosolic region. From the perspective of PIRB, mutants that maintain the HACS1 binding site but ablate the pYxxL site where SHP1 and SHP2 bind could be instructive.

While the interaction with PIRB receptor places HACS1 in signaling processes occurring at the cell membrane, HACS1 is also found in the nucleus where it has the potential to interact with components of the SAP30-HDAC chromosome remodeling complex^46^. Cortactin, an adaptor protein that is recruited to sites of actin reorganization, has also been identified as a ligand of HACS1 SH3 domain although the specific nature of this interaction has not been investigated further^47^. When all of the known and potential partnerships are considered, we believe that HACS1 has the potential to be a important signaling node in processes governing immune and neuronal cell fate.

## Methods

### Cloning, expression, and protein purification

A set of human HACS1 (UniProtKB: Q9NSI8, gene: SAMSN-1) clones were provided by Jaime Claudio (University of Toronto) as a starting point for deletion analysis. This set was used to make a number of SH3 domain (155–228) containing gene fragments that were cloned via the Gateway system into the expression vector pDEST-586 (NIH Structural Genomics Consortium). This vector appends an amino-terminal 6xHis tag, maltose-binding protein (MBP) tag, and a tobacco etch virus (TEV) protease recognition site. In later studies, the MBP-fusion set was replaced by codon-optimized, 6xHis-tagged HACS1 SH3 domain that was gene synthesized and provided in a pD441NH expression vector from ATUM (Newark, CA). Gene synthesis was also used to manufacture a 6xHis/Flag-tagged human SHP2 N-SH2 domain (UniProtKB: Q06124, gene: PTPN11, aa. 1–107) and a 6xHis/Protein G B1 domain tagged murine PIRB fragment (UniProtKB: P97484, gene: LILRB3, aa. 797–809) separated by a random 13 amino acid linker. Expression plasmids regulated by a T7 promoter were transformed into *E. coli* BL21(DE3) and plasmids regulated by a T5 promoter were transformed into *E. coli* BL21. A 1.5 L fermentation at 37 ˚C in a minimal medium containing 1 g of ^15^NH_4_Cl as the sole nitrogen source and 3 g of ^13^C-glucose as the sole carbon source was sufficient to manufacture a sample for NMR structural studies. Otherwise, fermentations were carried out in standard LB media. For all protein expression studies, a similar protocol was followed: Briefly, when the bacterial culture reached an A_600_ of 0.6, soluble protein expression was initiated by the addition of IPTG to 1 mM and the culture was incubated further for three hours. After centrifugation, the cell pellet was resuspended and lysed in a French pressure cell. The soluble 6x-His-tagged proteins were purified by affinity chromatography on a Nickel-NTA column (Qiagen), incubated with in-house made 6xHis tagged TEV protease to remove the MBP carrier protein if required, and purified further by gel filtration chromatography (Sephacryl-100, HiLoad 16/60; GE Biosciences). Unless otherwise indicated, proteins were used and stored in a buffer consisting of 20 mM sodium phosphate, pH 7.8, 0.15 M NaCl, 5 mM DTT, and 0.05% NaN_3_.

### Cell culture and pull downs

Mouse bone marrow and splenocyte lysates were prepared following a previously described method^15^. To 500 µg of lysate, 10 µg of purified 6xHis-MBP-HACS1 SH3 domain or 6xHis-MBP protein were mixed and incubated overnight with 50 µL Nickel-NTA resin. After washing and elution with TBST (20 mM Tris, 150 mM NaCl, 0.1% (w/v) Tween-20, pH 7.4), proteins were resolved on a 10% SDS polyacrylamide gel and transferred to a PVDF membrane. After blocking the membrane in TBST supplemented with 5% skimmed milk, it was probed with a mouse anti-PIRB antibody MAB2754 (R&D Systems; Minneapolis MN) and detected by chemiluminescence.

### Peptide array

A set of 12-mer peptides on a 150 × 100 mm cellulose membrane arranged in a 20 × 30 array were synthesized using the SPOT method^48^ with an Intavis MultiPep instrument. A crude estimate of the peptide content in each spot was made by staining the array with Fast Green FCF. The array was probed with 5 µM of 6xHis-MBP-HACS1-SH3 in PBST (3.2 mM sodium phosphate, 0.5 mM potassium phosphate, 1.3 mM KCl, 135 mM NaCl, 0.1% Tween-20, pH 7.4). Following blocking and washing with 5% skimmed milk and 2.5% bovine serum albumin (Bioshop; Burlington ON) in PBST, bound protein was identified by incubating the array with a 1:5000 dilution of horseradish peroxidase (HRP)-conjugated 6xHis monoclonal antibodies (Qiagen) in PBST and a chemiluminescent reagent (Santa Cruz Biotechnology).

### Surface plasmon resonance binding assay

A streptavidin-derivatized gold sensor chip for an OpenSPR instrument (Nicoyalife; Waterloo ON) was coated with either biotinylated human (GGQDVT{pY}AQLHSLTRLREATE) or murine (GGQDVT{pY}AQLCSRTLRQGAAAS) PIRB derived phosphopeptide (Genscript; Piscataway, NJ). Pure, 6xHis-tagged HACS1 SH3 domain protein or 6xHis-tagged human SHP2 N-SH2 domain protein was prepared at concentrations ranging from 20 µM to 0.1 µM in analyte buffer (10 mM Tris-HCl, 100 mM NaCl, pH 7.4, 0.1% bovine serum albumin, 0.005% Tween-20, 0.05% NaN_3_). This analyte buffer was selected to minimize nonspecific binding effects. The addition of either competitor protein or detergent alone did not mitigate nonspecific binding effects completely. Flow rates for binding and washing phases of the experiment were performed at 40 µL/min. The chip was regenerated after each binding experiment with 10 mM HCl. Data were processed with TraceDrawer (Ridgeview Instruments; Vȧnge Sweden) and exported to proFit (Quansoft; Uetikion am See, Switzerland) for presentation.

### Differential scanning calorimetry (DSC)

A degassed protein sample at 2.0 mg/mL and matched buffer reference (5 mM Tris, 60 mM NaCl, 2.5 mM TCEP pH 7.7) were loaded into a Nano DSC (TA Instruments) and subjected to a temperature gradient from 10 to 95 °C at 1 °C/min at 3 atm pressure. Background data were subtracted from a run where the buffer was used in both the sample and reference cells. The DSC data were best fit to a two-state scaled model with one transition.

### Size-exclusion chromatography with multiangle laser scattering (SEC-MALS)

Protein samples were prepared in the same manner as for the DSC experiments. The chromatography system was configured with an Infinity-II HPLC (Agilent) and AdvanceBio SEC300A column (Agilent). To ensure optimal stability, the chromatography system was equilibrated with the sample buffer for 18 h before the first injection. The detection system consists of a MiniDAWN TREOS MALS and OptiLab T-rEX refractive index module (Wyatt). Prior to the analysis, a 2.0 mg/mL bovine serum albumin standard was injected to calibrate the peak and retention time characteristics of the flow cells. Following calibration, a 20 µL HACS SH3 domain protein sample at 2.0 mg/mL was injected. Chromatograms were analyzed and molecular masses determined with ASTRA software (Wyatt).

### Isothermal titration calorimetry (ITC)

Measurements were performed on VP-ITC calorimeter (MicroCal, Northampton, MA). An optimal titration was observed when 37 μM 6xHis-tagged HACS1 SH3 domain was in the sample cell and a 600 μM solution of PIRB-GB1 was in the syringe. Prior to the experiment, proteins were exchanged by gel filtration chromatography into identical buffers (10 mM Tris, 50 mM NaCl, 1 mM TCEP, 0.05 NaN_3_, pH 7.5). After the first injection of 2 μL, the titration followed with successive injections of 8 μL with an equilibration delay of 300 s. The heat of dilution was determined by titrating the PIRB-GB1 solution into buffer alone. The raw data were corrected for heat of dilution and fitted to a single-site binding model using Origin v5.0 (OriginLabs, Northampton MA).

### Nuclear magnetic resonance (NMR) spectroscopy

To assign and determine the structure of the HACS1 SH3 domain, a conventional heteronuclear, triple-resonance strategy was followed using datasets acquired at 25 ˚C on an Agilent 600 MHz NMR spectrometer equipped with a helium-cooled probe and a uniformly ^15^N, ^13^C-labeled sample of the HACS1 SH3 domain at 0.6 mM in a buffer consisting of 20 mM sodium phosphate pH 7.7, 0.15 M NaCl, 5 mM DTT, 0.05% NaN_3_, and 10% D_2_O. A pH of 7.7 was selected for protein optimal solubility. Specific NMR experiments include HNCACB, CBCA(CO)NH, HNCO, HNCACO for backbone assignments, H(C)(CO)NH, C(CO)NH, HCCH-TOCSY, HB(CBCG)CD, HB(CBCGCD)CE for side-chain assignments and ^15^N-edited ^1^H-^1^H NOESY and ^13^C-edited ^1^H-^1^H NOESY experiments (100 ms mixing time) for distance restraints. All assignments were made manually. Amide residual dipolar couplings from IPAP-HSQC spectra were acquired using a ^15^N-labeled protein sample supplemented with 10 mg/mL Pf1 phage (Asla-Biotech, Riga Lativa). Binding experiments using ^15^N-labeled HACS1 SH3 domain and Protein GB1-tagged PIRB were acquired on a Bruker Avance 700 MHz instrument equipped with a nitrogen chilled probe. All datasets were processed with NMRPipe^49^ and interpreted with CCPN Analysis^50^. A relaxation study at 700 MHz was performed to determine ^15^N T_1_ relaxation times (60, 100, 200, 400, 600, 800, 1000 ms delays), ^15^N T_2_ relaxation times (10, 30, 50, 70, 90, 110, 130, 150 ms delays) and heteronuclear ^15^N{^1^H}NOEs. Using a freshly prepared and reduced 0.3 mM sample of ^15^N-labeled in a similar NMR buffer except DTT was replaced with 2.5 mM TCEP. 6xHis-tagged HACS1 SH3 domain, relaxation data were acquired as interleaved pseudo-3D spectra. Peak integrations were made with the nLinLS module of NMRPipe, followed by curve fitting to an exponential decay function with LMQUICK^51^. A correlation time was calculated by a global analysis of the relaxation data^52^.

### Structure determination

NOE distances were calculated from peak volumes with CCPN Analysis. Backbone torsion angles were predicted from chemical shifts using TALOS-N^53^. From an ensemble of 500 structures calculated with CYANA^54^, the top structure was selected among twenty that had no NOE violations > 0.2 Å and no torsion angle violations > 5˚. This structure served as input for refinement with XPLOR-NIH v2.58^55^. The supplied *refine.py* script was used as supplied except the maximum force constant for the RDC energy term was set to 0.2 kcal•mol-^1^•Hz-^1^. Inferred hydrogen bonds from backbone NOEs were explicitly defined for the HBDA energy term^56^ and other hydrogen bonds were determined during the refinement by the HBDB energy term^57^. From 400 structures, a final ensemble of twenty structures was selected based on the lowest energy and no NOE violations > 0.2 Å and no torsion angle violations > 5˚. Structure quality was assessed by PSVS^58^ and PROCHECK^59^. From a Ramachandran analysis of residues 167–174,184–196, 202–207, 210–214, and 216–221 in the ensemble, 93.4% residues were located in the most favored region, 6.5% were located in additional allowed regions, 0.1% were located in generously allowed regions, and no residues were located in disallowed regions.

### Chemical shift mapping

A ^1^H,^15^N-HSQC spectrum was acquired with a sample of ^15^N-labeled HACS1 SH3 domain in a matched NMR buffer containing an excess Protein G B1 domain tagged PIRB. A weighted chemical shift perturbation, *d*, was calculated using the following relationship^60^.

$$d = \sqrt {\frac{1}{2}\left[ {\delta _H^2 + \left( {0.14 \cdot \delta _N^2} \right)^2} \right]}$$

### SHP2-PIRB molecular model

The coordinates of the SHP2 SH2-N domain crystal structure (PDB: 4QSY) bound to a GAB1 phosphopeptide was used as a starting point to make a molecular model of the SH2 domain bound to PIRB ITIM3. The GAB1 peptide was extended several amino acids with SWISS-MODEL^61^ and then modified to match PIRB with Rosetta Remodel^62^.

### Bioinformatics

To identify potential structured and intrinsically disorder regions of the murine PIRB cytosolic domain, the protein sequence was analyzed by Quick2D utility of HHPRED^63^. Specific secondary structure prediction programs used were PSIPRED^39^, SPIDER3^40^, and DeepCNF-SS^41^. Specific intrinsically unstructured region prediction programs used were IUPred^36^, SPOT-Disorder^37^, and DISOPRED3^38^.

### Statistics and reproducibility

NMR solution structure statistics are expressed as the root-mean-square deviation from the average structure or value.

### Reporting summary

Further information on research design is available in the Nature Research Reporting Summary linked to this article.

## Supplementary information

Supplementary Information

Reporting summary

## Data Availability

The datasets generated and analyzed during the current study are available in the Protein Data Bank (accession code 6UZJ) and the BioMagResBank (accession code 30684). Original data for Fig. 4, Fig. 6, and Supplementary Figure S5 are available at the YorkSpace Institution Repository (http://hdl.handle.net/103015/38713). All other data are available upon request.
