# Supplementary material for: HACS1 signaling adaptor protein recognizes a motif in the paired immunoglobulin receptor B cytoplasmic domain
Source: Commun Biol. 2020 Nov 13;3:672. doi: 10.1038/s42003-020-01397-z (PMC7666139; doi:10.1038/s42003-020-01397-z)
Supplement: Supplementary file 1 — Supplementary Information [file 42003_2020_1397_MOESM1_ESM.pdf]

Supplementary Information

HACS1 signaling adaptor protein recognizes a motif in the Paired Immunoglobulin Receptor B cytoplasmic domain

Jamie J Kwan, Sladjana Slavkovic, Michael Piazza, Dingyan Wang, Thorsten Dieckmann, Philip E. Johnson, Xiao-Yan Wen, and Logan W Donaldson

Table S1	Chemical shift assignment summary for the HACS1 SH3 domain
Figure S1	Sequence alignment of murine and human HACS1
Figure S2	SEC-MALS study of the HACS1 SH3 domain
Figure S3	Graphical summary of backbone and side chain assignments
Figure S4	Scatter plot of observed and expected residual dipolar couplings
Figure S5	NMR relaxation analysis of the HACS1 SH3 domain
Figure S6	Differential scanning calorimetry of the HACS1 SH3 domain
Figure S7	Western detection of the HACS1-PIRB interaction from human tissue
Figure S8	Isothermal titration calorimetry binding study of PIRB-ITIM3 with the HACS1 SH3 domain
Figure S9	Sequence analysis of murine PIRB

Category	Available	Assigned	% assigned
Element C	300	257	85.7
Element H	367	341	92.9
Element N	79	61	77.2
Backbone + H + HA	307	295	96.1
Backbone	183	173	94.5
Side chain H	243	219	90.1
Side chain non-H	196	145	74.0

Table S1 — Chemical shift assignment summary for the HACS1 SH3 domain (aa. 27-87). The first twenty-six amino acids are unassigned. This table was produced by CCPN Analysis v2.4.

```
Q9NSI8 mHACS1   MLKRKPSNVSEKEKHQKPKRSSSFGNFDRFRNNSLSKPDDSTEAHEGDPTNGSGEQSKTS
P57725 hHACS1   MLKRKPSNASDKEKHQKPKRSSSFGNFDRFRNNSVSKSDDSIEVHDRELTNGSEEQSKTS

Q9NSI8 mHACS1   NNGGGLGKKMRAISWTMKKKVGKKYIKALSEEKDEEDGENAHPYRNSDPVIGTHTEKVSL
P57725 hHACS1   SSGGSLGKKVRAISWTMKKKVGKKYIKALSEEKEEESGEEALPYRNSDPMIGTHTEKISL

                                                                     SH3
Q9NSI8 mHACS1   KASDSMDSLYSGQSSSSGITSCSDGTSNRDSFRLDDDGPYSGPFCGRARVHTDFTPSPYD
P57725 hHACS1   KASDSMDSLYSGQSSSSGITSCSDGTSNRDSFRLDDDSPYSGPFCGRAKVHTDFTPSPYD

Q9NSI8 mHACS1   TDSLKIKKGDIIDIICKTPMGMWTGMLNNKVGNFKFIYVDVISEEEAAPKKIKANRRSNS
P57725 hHACS1   TDSLKIKKGDIIDIICKTPMGMWTGMLNNKVGNFKFIYVDVILEEEAAPKKIKVPRSRRR

                                                                     SAM
Q9NSI8 mHACS1   KKSKTLQEFLERIHLQEYTSTLLLNGYETLEDLKDIKESHLIELNIENPDDRRRLLSAAE
P57725 hHACS1   ENHQTIQEFLERIHLQEYTSTLLLNGYETLDDLKDIKESHLIELNIADPEDRARLLSAAE

Q9NSI8 mHACS1   NFLEEEIIQEQENEPEPLSLSSDISLNKSQLDDCPRDSGCYISSGNSDNGKEDLESENLS
P57725 hHACS1   SLLDEETTVEHEKESVPLSSNPDI-LSASQLEDCPRDSGCYISSENSDNGKEDLESENLS

Q9NSI8 mHACS1   DMVHKIIITEPSD
P57725 hHACS1   DMVQKIAITESSD
```

Figure S1 — Sequence alignment of murine and human HACS1. Each protein is also referenced by its Uniprot code. Shading indicates identity. The SH3 and SAM domains are indicated.

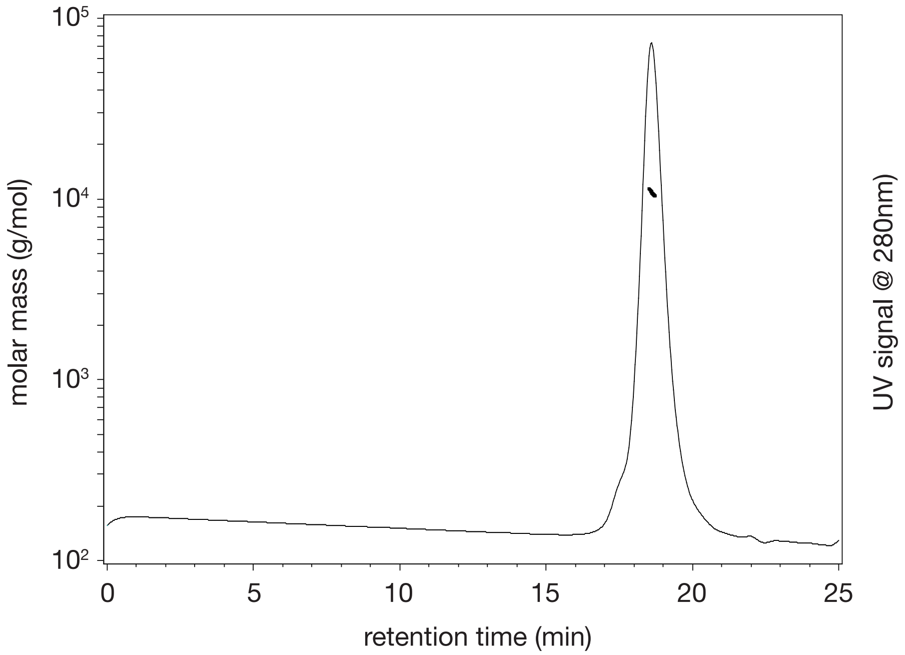

Figure S2 — Size exclusion chromatography with multi-angle laser scatter (SEC-MALS) analysis. 20 uL of 2 mg/mL HACS1 SH3 domain was subjected to chromatography (mobile phase: phosphate buffered saline) and detection by UV absorbance, MiniDAWN TREOS MALS, and OptiLAB T-rEX refractive index instruments. After data processing, the the observed peak coincides with a molecular mass of 10.9 ± 0.3 kDa suggesting that the HACS1 SH3 domain is monomeric (expected molecular mass, 10.65 kDa).

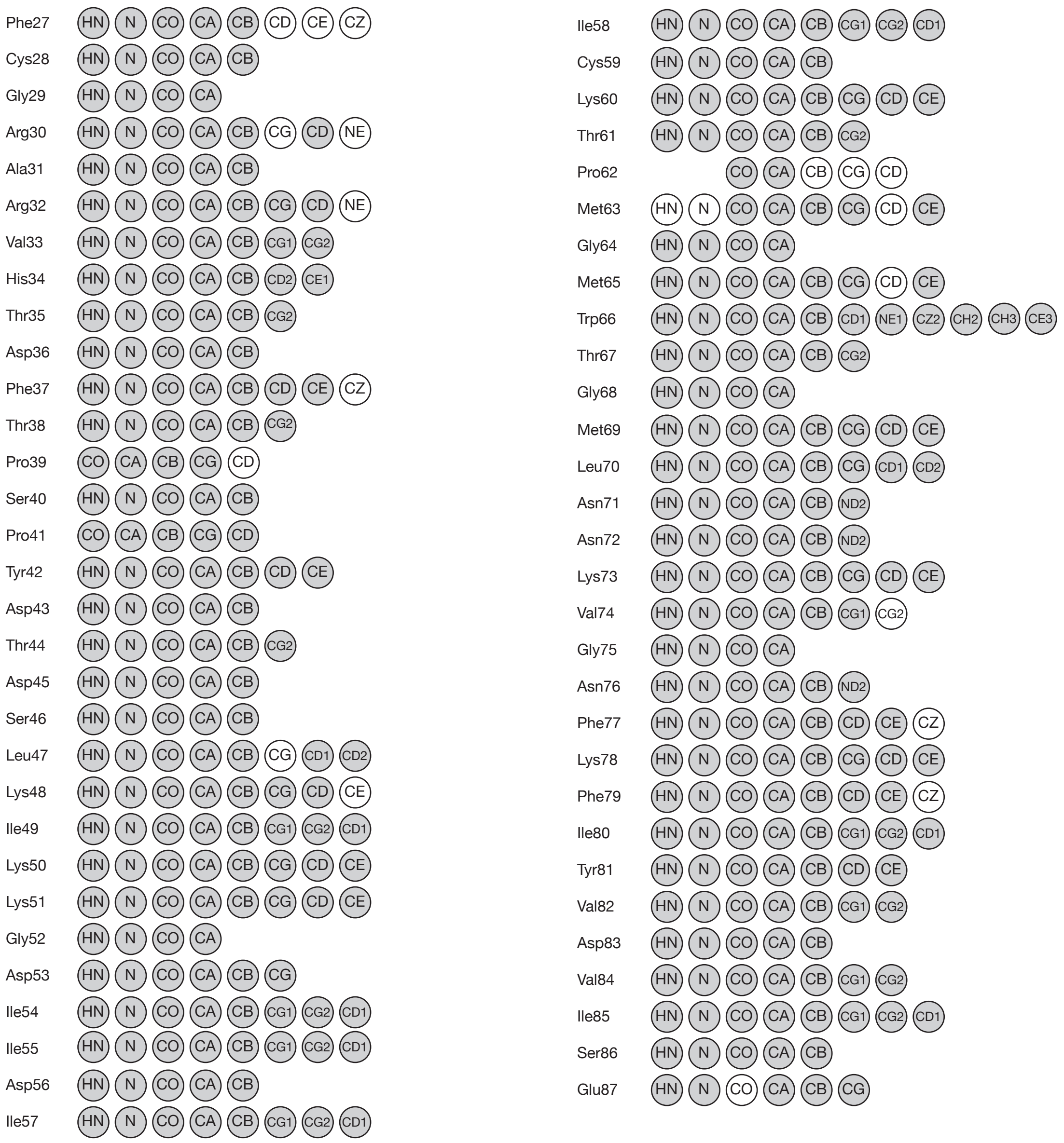

Figure S3 — Graphical summary of backbone and side chain heavy atom assignments for the HACS1 SH3 domain. The first twenty-six amino acids of the protein fragment are unassigned. Shading indicates that an assignment was made.

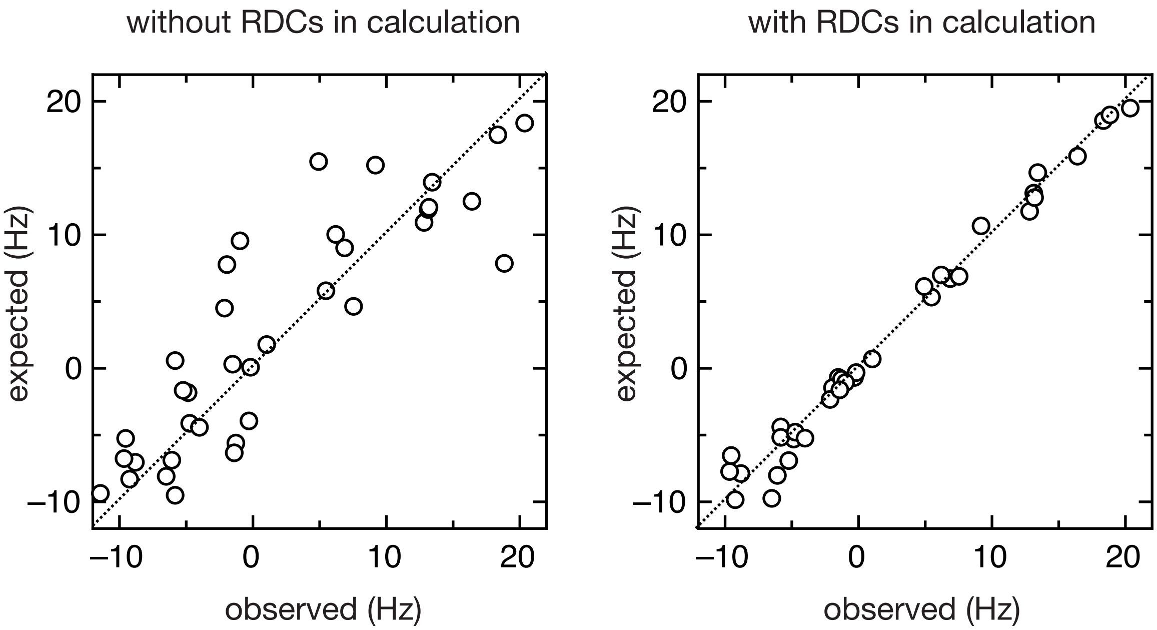

Figure S4 — Scatter plot of observed and expected HN residual dipolar couplings (RDCs) for the lowest energy structure of an ensemble that was calculated without or without the RDC dataset (*n*=42). Statistics are presented in Table 1 of the main text.

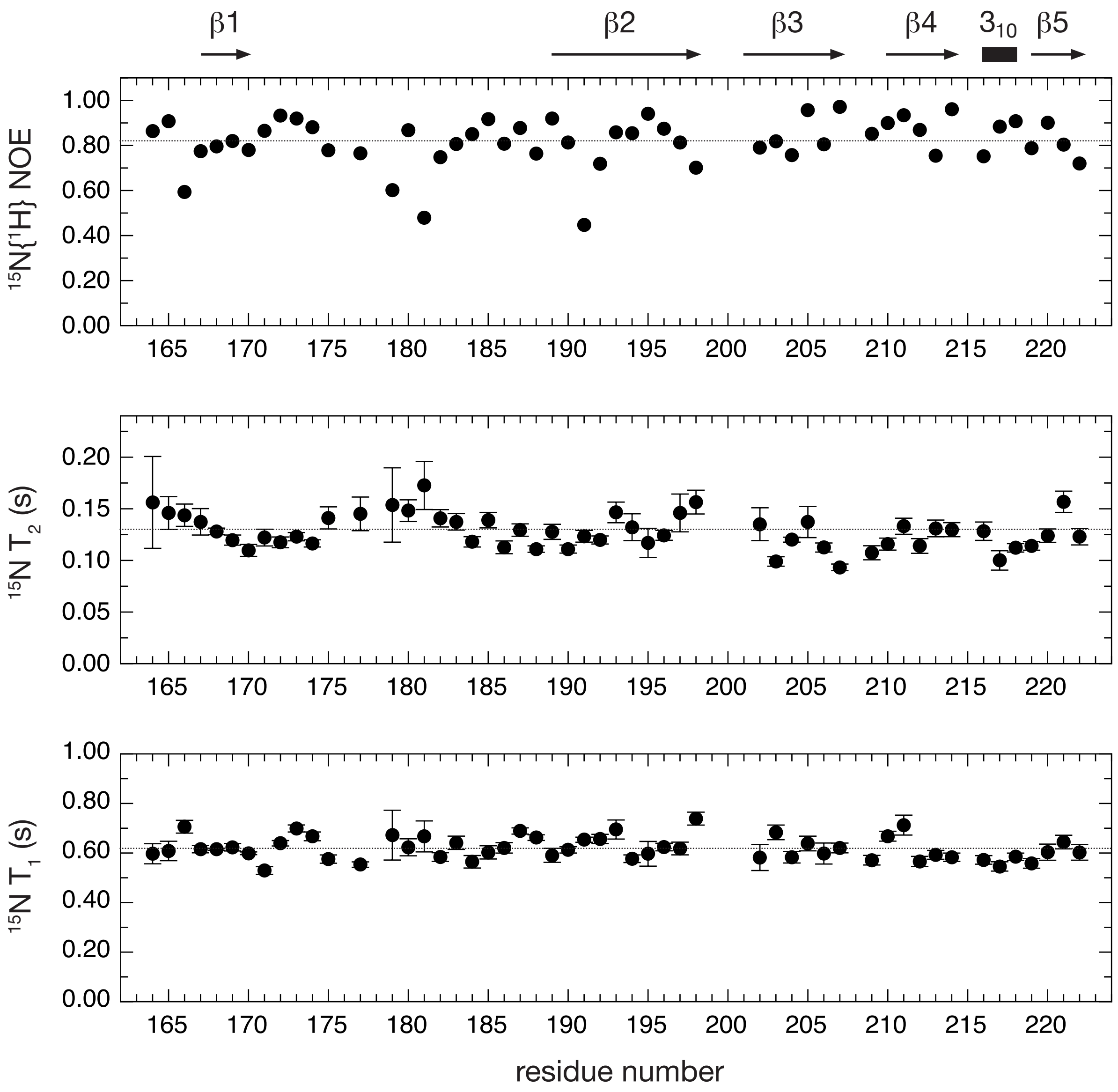

Figure S5 — NMR relaxation analysis of the HACS1 SH3 domain. Data were acquired at 25°C on a 700 MHz spectrometer. Observed secondary structures are indicated above the graphs for reference. A dotted line indicates the average value. Error bars indicate the standard deviation of the fit to a monoexponential rate.

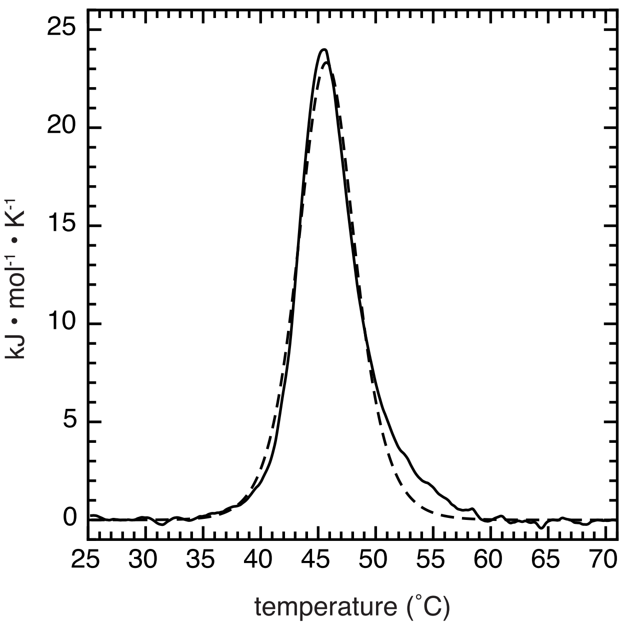

Figure S6 — Differential scanning calorimetry of the HACS1 SH3 domain. The unfolding was not reversible with a thermal transition midpoint (T_m) of 47.5 ˚C.

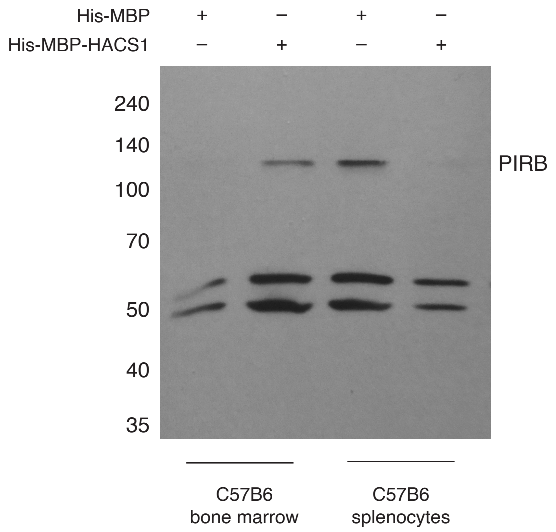

Figure S7 — Western detection of the HACS1-PIRB interaction. Immunoprecipitations: Lanes 1 and 2: C57B6 mouse bone marrow protein lysate with 6xHis-MBP control protein and 6xHis-MBP-HACS1-SH3 protein, respectively. Lanes 3 and 4: C57B6 mouse splenocyte protein extract with 6xHis-MBP-HACS1-SH3 protein and 6xHis-MBP control protein, respectively.

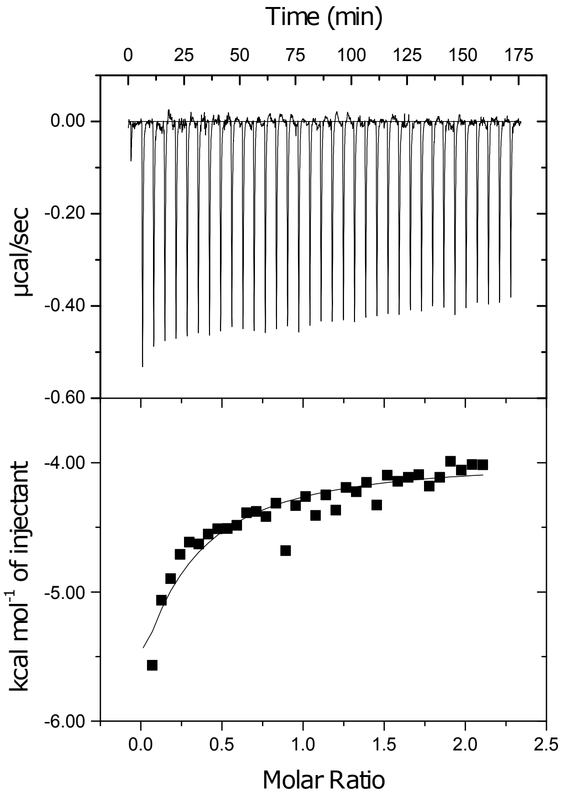

Figure S8 — Isothermal titration calorimetry. A reaction cell containing 91 uM HACS1 SH3 domain in 20 mM Tris pH 7.9, 50 mM NaCl, 1 mM TCEP was titrated with a 1.0 mM murine PIRB ITIM3 sequence fused amino-terminally to the Protein G B1 domain for enhanced solubility. This figure represents data that were adjusted for heat of dilution. The data were fit to 1:1 binding equilibrium with K_D of 15.9 uM.

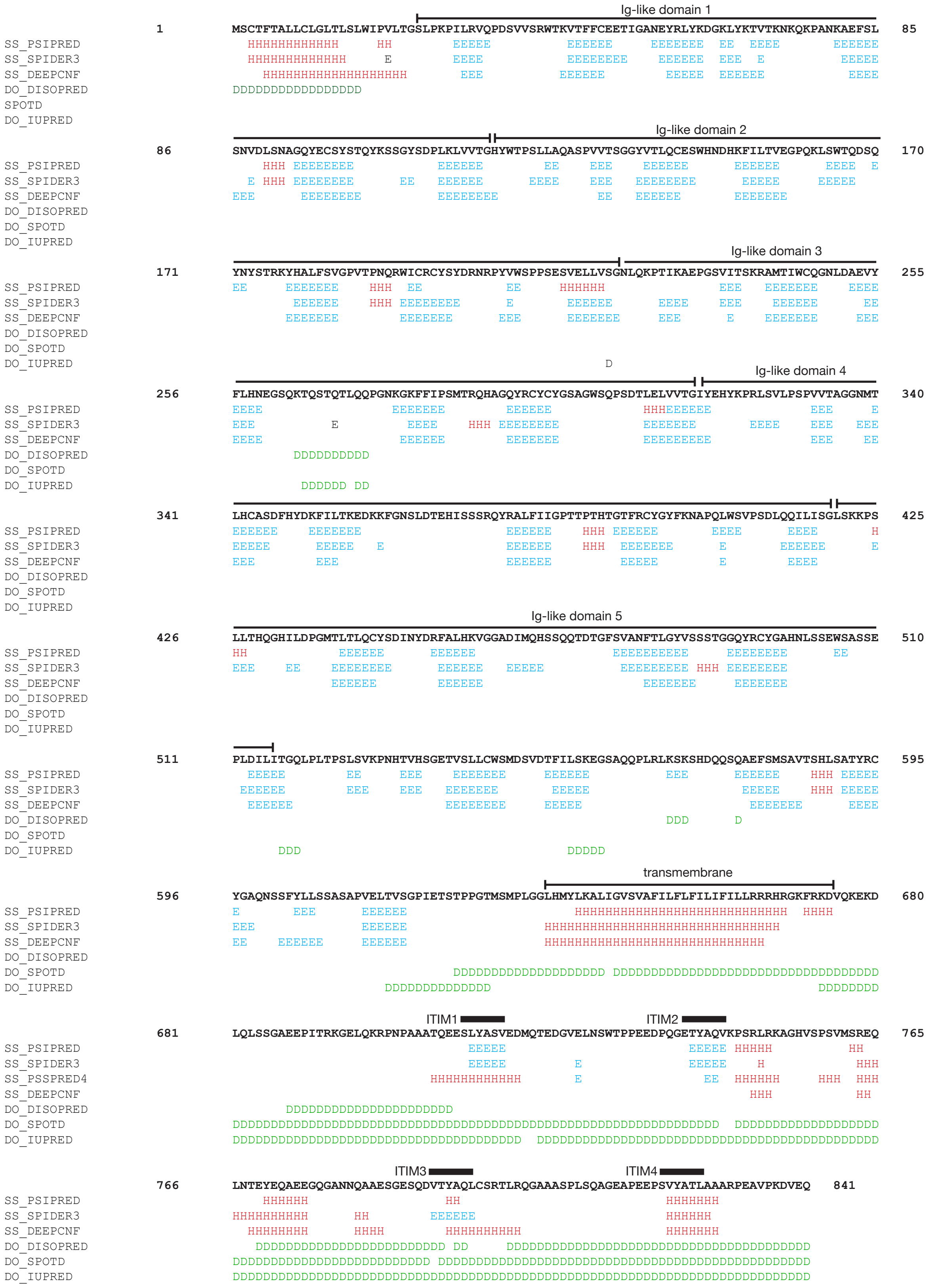

Figure S9 — Sequence analysis of murine PIRB. Helical (H), strand (S) and disordered (D) regions were predicted by PSIPRED (Buchan & Jones. 2019. Nucl Acids Res 47: W402), SPIDER3 (Heffernan *et al*. 2017. Bioinformatics 33:2842), DEEPCNF (Wang *et al.* 2016. Sci Rep 11:18962), DISOPRED3 (Jones & Cozzetto. 2015. Bioinformatics 31: 857), SPOTD (Hanson *et al.* 2017. Bioinformatics 33: 685) and IUPRED (Dosztányi *et al.* 2005. J Mol Biol 347: 827). The Ig repeats, transmembrane region, and ITIMs 1-4 are indicated. Ig-like domain boundaries are derived from the PIRB extracellular domain crystal structure (PDB:6GRQ; Vlieg et al. 2019. J Biol Chem 294: 4634).
